# Does the use of paclitaxel or rapamycin-eluting stent decrease further need for coronary-artery bypass grafting when compared with bare-metal stent?

**DOI:** 10.1590/S1516-31802007000400010

**Published:** 2007-07-01

**Authors:** Hernani Pinto de Lemos, Álvaro Nagib Atallah

**Keywords:** Angina pectoris, Coronary artery bypass, Percutaneous transluminal coronary angioplasty, Myocardial revascularization, Thoracic surgery, Angina pectoris, Ponte de artéria coronária, Angioplastia transluminal percutânea coronária, Revascularização miocárdica, Cirurgia cardíaca

## Abstract

**CONTEXT AND OBJECTIVE:**

The safety and efficacy of drug-eluting stents reduce the need for surgical revascularization. The objective of the present study was to investigate whether paclitaxel or rapamycin-eluting stent are effective in avoiding the need for coronary-artery bypass grafting.

**METHODS:**

This was a systematic review of the literature using the methodology of the Cochrane Collaboration. The type of study considered was controlled randomized trials; the type of intervention was drug-eluting or bare-metal stents; and the main outcome investigated was coronary-artery bypass grafting.

**RESULTS:**

The ten studies included in this systematic review did not show any statistically significant difference between the drug-eluting stents and the bare-metal stents with regard to the outcome of coronary-artery bypass grafting (confidence interval: 0.31 to 1.42).

**CONCLUSION:**

The surgical revascularization rate was not reduced by the use of drug-eluting stents.

## RESUMO

**CONTEXTO E OBJETIVO:**

A segurança e eficácia dos *stents* recobertos com drogas reduz a necessidade da cirurgia de revascularização cardíaca. O objetivo foi verificar se os *stents* recobertos com paclitaxel ou rapamicina são efetivos para evitar a necessidade de revascularização cirúrgica das coronárias.

**MÉTODOS:**

Revisão sistemática da literatura usando a metodologia do Centro Cochrane. Tipos de estudos: estudos clínicos randomizados; tipo de intervenção: *stents* recobertos com drogas ou *stents* convencionais; desfecho principal: cirurgia de revascularização cardíaca.

**RESULTADOS:**

Os 10 estudos incluídos nesta revisão sistemática não mostram diferença estatisticamente significante entre os *stents* recobertos com drogas e os *stents* convencionais no desfecho principal, com intervalo de confiança de 0.31, 1.42.

**CONCLUSÃO:**

A taxa de revascularização cardíaca não é reduzida pelo uso de *stents* recobertos com drogas.

## INTRODUCTION

Restenosis following stent implantation is a consequence of neointimal hyperplasia resulting from the proliferation and migration of smooth-muscle cells and production of extracellular matrix. The medical literature shows that drug-eluting stents are agents capable of interrupting cell replication and that stents show promise in inhibiting neointimal hyperplasia.^1-3^ There are two drugs commonly used: paclitaxel, a lipophilic molecule derived from the Pacific yew tree Taxus brevifolia that is capable of inhibiting cell division, motility, activation, secretory processes and signal transduction; and sirolimus (rapamycin), a cytostatic macrocyclic lactone with both anti-inflammatory and antiproliferative properties.^4^ Both restenosis and percutaneous revascularization are outcomes in all of the published studies. Drug-eluting stents appear to be a possibility for treating the coronary arterial disease, as a replacement for the existing therapies, but there is no proof of their effectiveness and safety. No differences in vital outcomes like mortality and myocardial infarction have been found between drug-eluting stents and bare-metal stents. However, such reports have not indicated whether there is any decrease in the need for coronary-artery bypass grafting (CABG).

## OBJECTIVE

The objective of the present study was to investigate whether paclitaxel or rapamycin-eluting stents are effective in avoiding the need for CABG.

## METHODS

The search strategy involved the following: a) online databases: Literatura Latino-Americana e do Caribe em Ciências da Saúde (Lilacs), Medical Literature Analysis and Retrieval System Online (Medline) and the Cochrane Library; b) manual search for studies; c) personal communication; and d) contact with the pharmaceutical industry. Two reviewers independently inspected the references of the studies found using this search strategy, and applied the inclusion criteria. Where disagreement occurred this was resolved by discussion or, if doubts remained, the full article was acquired for further inspection. An intention-to-treat analysis was done. The relative risks (RR) and their respective 95% confidence intervals (CI) were calculated based on the random effects model, since this takes into account any differences between studies, even if there is no statistically significant heterogeneity. The data were inspected to see whether analysis using a fixed effects model would make any substantive difference. The methodological quality of the trials selected was assessed using the criteria described in the Cochrane Handbook.^5^ The Jadad scale^6^ was also used.

### Inclusion criteria

**Type of study:** Only randomized controlled trials were considered. The adequacy of allocation concealment was assessed as shown in Table 1. Only studies from categories A and B were considered for inclusion.

**Table 1.** Adequacy of allocation concealment of randomized controlled trial included

Risk of Bias	Interpretation	Relationships to individual criteria
A. Low risk of bias	Plausible bias unlikely to seriously alter the results	All of the criteria met
B. Moderate risk of bias	Plausible bias that raises some doubt about the results	One or more criteria partly met
C. High risk of bias	Plausible bias that seriously weakens confidence in the results	One or more criteria not met

**Type of participants:** patients with restenosis post-stent implantation.

**Type of interventions:** drug-eluting stents versus bare-metal stents.

**Type of outcome:** coronary artery bypass grafting.

After locating all of the eligible studies, the data were summarized in a meta-analysis. This systematic review was approved by the ethics committee of Universidade Federal de São Paulo.

### Included studies

The search strategy found 329 studies. Of these, 10 studies met the inclusion criteria that had been established. The excluded studies did not meet one or more of the inclusion criteria, particularly with regard to randomization and outcome. There was total agreement between the reviewers.

**Studies on paclitaxel-eluting stents:** Taxus I (Grube et al.),^7^ Taxus II (Colombo et al.),^8^ Taxus IV (Stone et al.),^9^ European evaLUation of pacliTaxel-Eluting Stent [ELUTES] (Gershlick et al.),^10^ Asian Paclitaxel-Eluting Stent [ASPECT] (Park et al.),^11^ and RX ACHIEVE Drug-Eluting Coronary Stent System In the Treatment of Patients with De NoVo NativE CoronaRy Lesions [DELIVER].^12^

**Studies on sirolimus (rapamycin)-eluting stents:** Randomized study with the sirolimus-eluting Velocity Balloon-Expandable Stent [RAVEL] (Morice et al.),^13^ SIRolImUS-Eluting Stent in De Novo Native Coronary Lesions [SIRIUS] (Moses et al),^14^ Canada-SIRolImUS-Eluting Stent in De Novo Native Coronary Lesions [C-SIRIUS] (Schampaert et al.),^15^ and Europe-SIRolImUS-Eluting Stent in De Novo Native Coronary Lesions [E-SIRIUS] (Schofer et al.).^16^

## RESULTS

**Figure 1.**
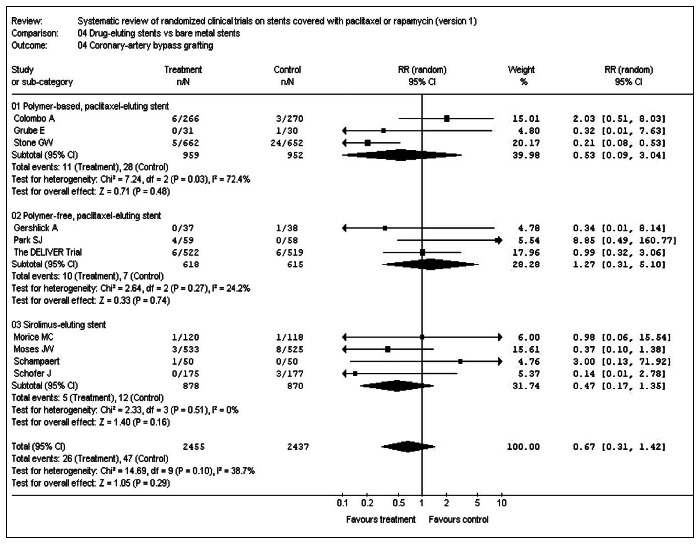
Meta-analysis — coronary-artery bypass grafting.

With regard to the outcome "need for coronary bypass grafting", the analysis was done in three subgroups: 1) polymer-based paclitaxel-eluting stents; 2) polymer-free paclitaxel-eluting stents; 3) sirolimus (rapamycin)-eluting stents. There were 2,455 participants in the experimental group and 2,437 in the control group. In total, 10 included studies reported this outcome. Only one study, Taxus IV, was favorable to the experimental group. The data from each study were analyzed using the RevMan software.^17^ The resulting meta-analysis did not find any statistically significant difference between the drug-eluting stents and the bare-metal stents: relative risk = 0.67; confidence interval: 0.31 to 1.42 (Figure 1 – RevMan – Statistical analysis).

The following other outcomes were also analyzed in this study:

**Thrombosis:** ten studies; none of them found any statistical difference for any group. Meta-analysis: relative risk = 1.1; confidence interval: 0.48 to 2.12.

**Mortality:** ten studies; none of them found any statistical difference for any group. Meta-analysis: relative risk = 1.23; confidence interval: 0.70 to 2.17.

**Myocardial infarction:** ten studies; none of them found any statistical difference for any group. Meta-analysis: risk relative = 0.84; confidence interval: 0.61 to 1.17.

**Percutaneous revascularization:** ten studies; seven favorable to the experimental group and three without any statistical difference for any group. Meta-analysis: relative risk = 0.32; confidence interval: 0.22 to 0.48; favorable to the experimental group.

**Restenosis:** ten studies; seven favorable to the experimental group and three without any statistical difference for any group. Meta-analysis: relative risk = 0.30; confidence interval: 0.21 to 0.43; favorable to the experimental group.

## DISCUSSION

This systematic review shows that there is no advantage in eluting stent over bare-metal stent. The literature shows that the trauma to the vessel wall that is caused by stent implantation triggers an inflammatory endothelial response, independent of the presence or absence of a drug in this stent. This initial inflammatory process is followed by a restenosis process.^18^ Comparison between balloon angioplasty and coronary stenting shows that the inflammatory process is less severe in balloon angioplasty, while in coronary stenting early neutrophil recruitment is followed by prolonged and abundant recruitment of macrophages within the neointima.^19,20^ After stenting, several inflammatory markers are released^21-23^ and their presence is associated with subsequent poor prognosis.^24^ Adverse late clinical outcomes are linked with the magnitude of the systemic inflammation. Patients may be risk-stratified according to their concentrations of inflammatory markers.^25^ Endothelial function becomes altered, thereby generating cytokine hypersecretion that preserves the inflammatory process and leads to changes in the quantities of endothelial mediators released, which may decrease the nitric oxide levels (vasodilatation) or increase the endothelin-1 levels (vasoconstriction).^26,27^ Studies have shown that drug-eluting stents may exacerbate the inflammatory process, such that they may accentuate the restenosis process at the stent extremities^28^ and boost the strength of some platelet agonists, thus promoting thrombus formation.^29^ Moreover, it has been demonstrated that sirolimus (rapamycin) reduces the production of nitric oxide.^30^

All the transformations following stent implantation may persist for months and years. Clinically, there may or may not be any symptoms, but the important point is that these alterations exist and impair the clinical treatment. Consequently, it can be deduced that surgical revascularization in patients who have already been revascularized with stents, and whose hearts present chronic inflammatory processes, will not have the same result as would be achieved in a heart that had never previously been revascularized with stents. In spite of the significant differences in all restenosis measurements, which were favorable to paclitaxel and rapamycin-eluting stents, the surgical revascularization rate was similar in the experimental and control groups.

This was confirmed at the recent World Congress of Cardiology held in Spain in September 2006. A meta-analysis with four years of follow-up was presented at that congress^31,32^ (the published studies included in our systematic review had one-year follow-ups), in which the outcomes were similar to those presented in our systematic review, with the addition of other data that suggested that there was increased noncardiac mortality and a higher cancer rate in the experimental group. These poor outcomes confirm all of the inflammatory alterations that originate from stent implantation and their persistent disastrous consequences.

Taking all these facts together, it has to be accepted that the clinical benefits from drug-eluting stents with regard to target vessel restenosis and target vessel revascularization have been overestimated and that they do not offer safety, efficacy and effectiveness.

## CONCLUSION

In the present study, paclitaxel or rapamycin-eluting stent did not show any statistically significant difference with regard to avoiding the need for coronary-artery bypass grafting, or in relation to myocardial infarction or death from cardiac causes, in comparison with bare-metal stent.
